# Isolation and characterization of a VHH targeting the *Acinetobacter baumannii* cell surface protein CsuA/B

**DOI:** 10.1007/s00253-023-12594-1

**Published:** 2023-06-07

**Authors:** Eric K. Lei, Shannon Ryan, Henk van Faassen, Mary Foss, Anna Robotham, Isabel Baltat, Kelly Fulton, Kevin A. Henry, Wangxue Chen, Greg Hussack

**Affiliations:** 1 Human Health Therapeutics Research Centre, National Research Council Canada, Ottawa, Ontario Canada; 2 Department of Biochemistry, Microbiology and Immunology, University of Ottawa, Ottawa, Ontario Canada; 3 Department of Biology, Brock University, St. Catharines, Ontario Canada

**Keywords:** *Acinetobacter baumannii*, Antimicrobial resistance, CsuA/B, Nanobody, Outer membrane vesicle, Pilus, Single-domain antibody, VHH

## Abstract

**Abstract:**

*Acinetobacter baumannii* is a Gram-negative bacterial pathogen that exhibits high intrinsic resistance to antimicrobials, with treatment often requiring the use of last-resort antibiotics. Antibiotic-resistant strains have become increasingly prevalent, underscoring a need for new therapeutic interventions. The aim of this study was to use *A. baumannii* outer membrane vesicles as immunogens to generate single-domain antibodies (VHHs) against bacterial cell surface targets. Llama immunization with the outer membrane vesicle preparations from four *A. baumannii* strains (ATCC 19606, ATCC 17961, ATCC 17975, and LAC-4) elicited a strong heavy-chain IgG response, and VHHs were selected against cell surface and/or extracellular targets. For one VHH, OMV81, the target antigen was identified using a combination of gel electrophoresis, mass spectrometry, and binding studies. Using these techniques, OMV81 was shown to specifically recognize CsuA/B, a protein subunit of the Csu pilus, with an equilibrium dissociation constant of 17 nM. OMV81 specifically bound to intact *A. baumannii* cells, highlighting its potential use as a targeting agent. We anticipate the ability to generate antigen-specific antibodies against cell surface *A. baumannii* targets could provide tools for further study and treatment of this pathogen.

**Key points:**

*•Llama immunization with bacterial OMV preparations for VHH generation*

*•A. baumannii CsuA/B, a pilus subunit, identified by mass spectrometry as VHH target*

*•High-affinity and specific VHH binding to CsuA/B and A. baumannii cells*

**Supplementary Information:**

The online version contains supplementary material available at 10.1007/s00253-023-12594-1.

## Introduction

The rates of antimicrobial resistance (AMR) in most bacterial pathogens continue to increase globally (OECD 2018). Despite recent advances in antimicrobial development against resistant pathogens, particularly β-lactamase inhibitors (Gonzalez-Bello et al. 2020), the level of AMR among ESKAPE pathogens continues to outpace antibiotic development efforts (Fair and Tor 2014). Thus, alternative strategies to target resistant pathogens are urgently needed to maintain our current standards of infectious disease care.

Recent efforts in antimicrobial development have started to explore the use of site-directed delivery of therapeutics to specifically target pathogens of interest (Cavaco et al. 2022; Yang et al. 2021). Directed delivery of therapeutic payloads has been pursued as a strategy primarily for cancer chemotherapy, where off-target toxicity is a significant concern (Birrer et al. 2019). The impact of systemic treatment with broad-spectrum antibiotics on the microbiome and implications for patient health has been a subject of increasing interest (Langdon et al. 2016). Additionally, if the effectiveness of currently available antibiotics continues to decrease, the development of novel and more potent antimicrobials may be hampered by off target toxicity. In this case, the use of site-directed therapeutics such as antibody–antibiotic conjugates (Lehar et al. 2015; Mariathasan and Tan 2017) may restrict high antimicrobial concentrations at the site of infection, decreasing dosing requirements as well as reducing off-target effects including disruption of the microbiota.

We set out to generate single-domain antibodies (VHHs) against surface-exposed *Acinetobacter baumannii* membrane proteins. *A. baumannii* is a Gram-negative bacterial pathogen that exhibits high levels of AMR (Gales et al. 2019) and is listed as a critical priority for the development of new antibiotics by the WHO (Tacconelli et al. 2018). Nosocomial infections by *A. baumannii* show intrinsic resistance to most classes of antibiotics, resulting in the use of carbapenems as first-line treatment followed by polymyxins or tigecyclines (Asif et al. 2018). However, resistance of *A. baumannii* to carbapenems is already at critical levels in several countries, and the prevalence of multidrug-resistant isolates for which no effective pharmacologic interventions are available is rising (Pormohammad et al. 2020). In light of this, there is an urgent need for novel and effective therapies for *A. baumannii* infection. Moreover, because the pathogen has risen to prominence as a public health risk only recently, relatively few molecular tools exist to investigate new therapeutic interventions such as site-directed antimicrobials.

We leveraged the propensity of *A. baumannii* to produce outer membrane vesicles (OMVs) to generate VHHs capable of targeting outer membrane antigens. *A. baumannii* OMVs contain proteins that directly disrupt mammalian cell functions and induce cell death (Tiku et al. 2021), are involved in gene transfer (Chatterjee et al. 2017), and play a role in bacterial nutrient acquisition (Dhurve et al. 2022). OMVs contain a heterogeneous mixture of membrane components, including membrane-anchored and membrane-associated proteins, which could be targeted by antibodies (Kwon et al. 2009). Previous studies have indicated that *A. baumannii* OMVs are immunogenic in mice (Huang et al. 2019) and elicit a proinflammatory response in HEp-2 cells (Jun et al. 2013).

Well-characterized targeting agents against *A. baumannii*, such as monoclonal antibodies and antibody fragments, are still largely unavailable. VHHs are 12–15 kDa single-domain binding proteins derived from camelid heavy-chain-only immunoglobulins (Igs) and consist of a single, unpaired, polypeptide chains that are amenable to phage display-based selection. The development of VHHs is generally simpler and more cost-effective compared to conventional IgGs (Harmsen and De Haard 2007) as the smaller size and robust biophysical properties (e.g., high thermal and chemical stability, aggregation resistance) of these molecules simplify expression, engineering, and chemical modification. In this work, we report the isolation of VHHs against *A. baumannii* OMV preparations as well as the identification of the target antigen for one VHH, OMV81. VHH OMV81 was shown to recognize CsuA/B, the main structural component of the Csu pilus.

## Materials and methods

### OMV preparation

*A. baumannii* OMV preparations were prepared essentially as described previously (McConnell et al. 2011). *A. baumannii* strains ATCC 19606, ATCC 17961, ATCC 17978, and LAC-4 (Table 1), hereafter referred to as “19606,” “17961,” “17978,” and “LAC-4,” respectively, were first streaked on brain heart infusion (BHI) agar plates and incubated at 37°C overnight. A 10-μL loop was then used to collect samples of colonies from each strain that were resuspended in a conical tube containing 5 mL of tryptic soy broth (TSB). Next, 2 mL of the suspension was used to inoculate 100 mL of TSB in a 500-mL baffled flask. The flasks were incubated at 37°C with shaking at 200 rpm for 17 h. The bacteria were then centrifuged at 11,000 × *g* for 20 min at 4°C, and the supernatant was transferred to a new centrifuge bottle and spun again as above. The supernatant was filtered through a 0.45-μm syringe filter followed by a 0.22-μm syringe filter. The filtered samples were ultracentrifuged at 126,000 × *g* for 3 h at 4°C and then aspirated using a serological pipette. The pellets were resuspended in 4 mL of phosphate buffered saline (PBS; 137 mM NaCl 137 mM NaCl, 2.7 mM KCl, 8 mM Na_2_HPO_4_, and 2 mM KH_2_PO_4_, pH 7.4). Total protein in each OMV sample was quantified using a Pierce BCA Protein Assay Kit (Thermo Fisher Scientific, Waltham, MA).

**Table 1:** Bacterial strains used in this study

Species	Strain	Purpose	Source
*A. baumannii*	ATCC 19606	OMV source, whole-cell ELISA, microscopy	ATCC
	ATCC 17961	OMV source, whole-cell ELISA, microscopy	ATCC
	ATCC 17978	OMV source, whole-cell ELISA, microscopy	ATCC
	LAC-4	OMV source, whole-cell ELISA, microscopy	Valentine et al. (2008)
*E. coli*	TG1	Phagemid library host, protein expression	Agilent
	BL21(DE3)	Protein expression, whole-cell ELISA	Lucigen/VWR

### OMV particle analysis

OMVs from *A. baumannii* strains 19606, 17961, 17978, and LAC-4 were normalized to 100 μg/mL in 100 μL of degassed PBS based on total protein content. The samples were then loaded onto a ZetaView Quatt NTA PMX-420 (Particle Metrix GmbH, Inning, Germany), roughly quantified, and then further diluted into degassed PBS to a particle concentration of approximately 50–200 per image. OMV samples were then analyzed for particle diameter using scattering at 488 nm. A total of 11 image frames were analyzed and averaged for each OMV prep.

### OMV size exclusion chromatography (SEC)

Approximately 300 μg of OMV from *A. baumannii* strain 19606 was diluted into 400 μL of PBS and applied to a PBS-equilibrated Superose 6 Increase 10/300 GL column (Agilent, Santa Clara, CA) connected to a 1260 Infinity LC System (Agilent). The absorbance at 210 nm was monitored. Fractions were collected every 0.5 mL starting at 5 mL. Fraction collection volumes were corrected for the void volume between the UV detector and the elution fraction collector of 1.692 mL. Fractions were directly coated (neat) in wells of microtiter plates for indirect ELISA (*see* below).

### Llama immunization

OMVs from *A. baumannii* strains 19606, 17961, 17978, and LAC-4 were mixed at 75 μg of total OMV protein per strain for a total of 300 μg of OMV protein for each of the four immunizations. Prior to injection, each preparation was filtered through two 0.22-μm syringe filters into individual tubes and confirmed by plating on BHI agar to be free of viable *A. baumannii*. Immunizations were performed at Cedarlane Laboratories (Burlington, Canada), and subcutaneous injections were done with either Complete Freund’s Adjuvant (CFA; MilliporeSigma, Burlington, MA) or Incomplete Freund’s Adjuvant (IFA; MilliporeSigma). The immunization schedule was based on successful llama immunization campaigns previously performed by our group (Hussack et al. 2018) and proceeded as follows: day 0, pre-immune serum collection and priming immunization (300 μg OMV + CFA); day 21, boost 1 (300 μg OMV + IFA); day 28, boost 2 (300 µg OMV + IFA); day 35, test bleed + boost 3 (300 µg OMV + IFA); and day 42, terminal bleed. Peripheral blood mononuclear cells (PBMCs) were obtained from whole blood by density gradient centrifugation, and serum was prepared by clotting and centrifugation as previously described (Baral et al. 2013).

### Serum fractionation

Pre-immune and terminal bleed sera (1 mL each) were dialyzed into sodium phosphate buffer (20 mM sodium phosphate, 150 mM NaCl, pH 7.0) and loaded on a 1 mL HiTrap Protein G column (Cytiva, Vancouver, Canada) connected to an ÄKTA FPLC protein purification system (Cytiva). The G1 (IgG2b) fraction was eluted with 100 mM citrate buffer, pH 3.5, and the G2 (IgG1) fraction was eluted with 100 mM glycine buffer, pH 2.7. Both the G1 and G2 fractions were immediately dialyzed into sodium phosphate buffer.

### Serum ELISAs

Immune responses to OMVs from *A. baumannii* strains 19606, 17961, 17978, and LAC-4 were measured using G1 (IgG2b) and G2 (IgG1) fraction polyclonal IgGs from pre-immune and terminal bleed sera by ELISA. Briefly, each of the four OMVs (0.38 μg) was coated in wells of a microtiter plate (Corning, Glendale, AZ) in PBS by incubating at 4°C for 72 h. Wells were blocked with 5% (w/v) skim milk in PBS for 1 h at 37°C. Subsequently, 10-fold serial dilutions of fractionated serum IgGs (100 μL/well in PBS) were incubated in wells for 1 h at room temperature. Wells were washed 3× with PBST (PBS, pH 7.4 containing 0.05% (v/v) Tween-20) followed by incubation with a 1:10,000 dilution of horseradish peroxidase (HRP)-conjugated anti-llama IgG (Cat#A160-100P, Bethyl Laboratories, Montgomery, TX) in PBST for 1 h at room temperature. Wells were washed as above and developed with 50 μL of High Sensitivity 3,3′,5,5′-tetramethylbenzidine (TMB) ELISA Substrate (Abcam, Cambridge, UK) for 5 min followed by the addition of 50 μL of 1 M phosphoric acid. The absorbance was measured at 450 nm.

### Phage display library construction and panning

Phage-displayed VHH library construction and panning were performed essentially as described previously (Hussack et al. 2012). Briefly, total RNA was isolated separately from approximately 5.0×10^7^ day 35 PBMCs and 5.0×10^7^ day 42 PBMCs using the QIAamp RNA Blood Mini Kit (Qiagen, Mississauga, Canada). Next, 30 μg of total RNA (15 μg from day 35 and 15 μg from day 42) was used as template for first-strand cDNA synthesis using the SuperScript^TM^ VILO^TM^ Master Mix (Invitrogen, Carlsbad, CA). Rearranged VHH coding sequences were PCR-amplified in two reactions using an equimolar mix of three framework region 1 (FR1)-specific sense primers (MJ1, 5′-GCCCAGCCGGCCATGGCCSMKGTGCAGCTGGTGGAKTCTGGGGGA-3′; MJ2, 5′-CAGCCGGCCATGGCCCAGGTAAAGCTGGAGGAGTCTGGGGGA-3′; and MJ3, 5′-GCCCAGCCGGCCATGGCCCAGGCTCAGGTACAGCTGGTGGAGTCT-3′) and one of two antisense CH2-specific primers (CH2, 5′-CGCCATCAAGGTACCAGTTGA-3′, and CH2b3, 5′-GGTACCTGTCATCCACGGACCAGCTGA-3′). The PCR products were electrophoresed in a 1% agarose gel, and the smaller of two major bands (approximately 850 bp and 600 bp), corresponding to the VHH-CH2 amplicon, and the single VHH-CH2b3 amplicon at ~600 bp were excised and gel-purified using the QIAquick Gel Extraction Kit (Qiagen). The purified DNA was re-amplified in a second PCR using 10 pmol each of a FR1-specific sense primer (MJ7, 5′-CATGTGTAGACTCGCGGCCCAGCCGGCCATGGCC-3′) and a FR4-specific antisense primer (MJ8, 5′-CATGTGTAGATTCCTGGCCGGCCTGGCCTGAGGAGACGGTGACCTGG-3′). The resulting amplicons were purified using a QIAquick PCR Purification Kit (Qiagen), digested with *Sfi*I (New England Biolabs, Ipswich, MA), purified again in the same manner and ligated into a *Sfi*I-digested pMED1 phagemid vector. The ligated DNA was purified again using a QIAquick PCR Purification Kit and used to electroporate electrocompetent *Escherichia coli* TG1 cells (Agilent) in electroporation cuvettes (Bio-Rad, Hercules, CA). Library cells were expanded and cryopreserved as previously described (Baral et al. 2013).

For panning, 2 mL of library cells was grown in 200 mL of 2xYT containing 100 μg/mL ampicillin and 2% (w/v) glucose until an OD_600_ of 0.4–0.5 was reached. Cells were subsequently superinfected with M13KO7 helper phage (New England Biolabs) for 1 h at 37°C. The cells were pelleted at 4°C, resuspended in 2×YT containing 100 μg/mL ampicillin and 50 μg/mL kanamycin, and incubated overnight at 37°C with 250 rpm shaking. The next day, cultures were centrifuged, and library phage particles in the culture supernatants were mixed with 1/5 volume of 20% polyethylene glycol 6000 containing 2.5 M NaCl, incubated on ice for 2 h and centrifuged at 17,000 × *g* for 30 min.

*A. baumannii* OMVs from strains 19606, 17961, 17978, and LAC-4 (5 μg/well) were passively adsorbed in wells of Nunc microtiter plates. Wells were blocked with PBS containing 4% skim milk for rounds 1 and 3 and with SuperBlock (Thermo Fisher Scientific) for round 2 for 2 h at 37°C. After blocking, library phage in PBS (3×10^11^ colony-forming units (cfu)/well) were added and incubated for 1 h at room temperature. Wells were washed 3× with 300 μL of PBST followed by 3× with 300 μL of PBS. Bound phage was eluted with 100 μL of 100 mM triethylamine, pH 11. After a 10 min incubation, the eluates were removed from wells and neutralized with 50 μL of 1 M Tris-HCl, pH 7.4, in a new tube. Phage outputs were amplified in *E. coli* TG1 cells for the next round of panning, and a total of three rounds of panning were conducted.

### Phage ELISA and DNA sequencing

Binding of amplified output phage from all rounds of panning to *A. baumannii* OMVs was assessed by polyclonal phage ELISA (Baral et al. 2013). Wells of microtiter plates were coated with OMVs from *A. baumannii* strains 19606, 17961, 17978, and LAC-4 as described above and then blocked with 4% skim milk in PBS. Ten-fold serial dilutions of amplified VHH-displaying phage (approximately 1×10^10^ to 1×10^4^ cfu) were added to the wells and incubated for 1 h at room temperature. Wells were washed 3× with PBST and bound phage were detected with HRP-conjugated anti-M13 secondary antibody (Cat#SC-53004HRP, Santa Cruz Biotechnology, Santa Cruz, CA) diluted 1:5,000 in PBS. After washing again 3× with PBST, wells were developed with High Sensitivity TMB ELISA Substrate for 5 min. The reaction was stopped with 1 M phosphoric acid, and absorbance was measured at 450 nm. For each of the four OMV preparations, 45 individual phage clones from round 2 and 45 clones from round 3 were screened by monoclonal phage ELISA, as described above. The VHH coding sequences of binding clones were obtained by Sanger sequencing.

### Expression and purification of soluble VHHs

DNAs encoding VHH sequences were cloned into the pSJF2H expression vector (Arbabi-Ghahroudi et al. 2009), and the resulting constructs were used to transform electrocompetent *E. coli* TG1 cells. The expressed VHHs contained C-terminal c-Myc and His_6_ tags. DNA encoding a variant of OMV81 bearing a free C-terminal Cys residue was cloned into the modified pET28a expression vector pMRo.BAP.H6 (Rossotti et al. 2019) and used to transform electrocompetent *E. coli* BL21(DE3) cells (Lucigen distributed by VWR, Mississauga, Canada). Transformed cells were plated on 2×YT agar plates containing 50 μg/mL kanamycin for pMRo.BAP.H6 or 100 μg/mL ampicillin for pSJF2H. A single colony was picked and grown in 3 mL of 2×YT antibiotic-containing medium overnight at 37°C with 250 rpm shaking. This culture was used to inoculate 1 L of antibiotic-containing 2×YT medium. Cells were grown to an OD_600_ of 0.6 and then induced using 100 μM isopropyl β-D-1-thiogalactopyranoside (IPTG) for pSJF2H and 10 μM IPTG for pMRo.BAP.H6. Cells were then incubated for 24 h at 37°C with 250 rpm shaking, pelleted by centrifugation and lysed *via* sonication in buffer A (10 mM HEPES, 20 mM imidazole, 500 mM NaCl, pH 8.0). After filtration through 0.45-μm filters, the lysates were passed over a HiTrap immobilized metal affinity chromatography (IMAC) HP column (Cytiva) connected to an ÄKTA FPLC protein purification system, and bound VHHs were eluted using a linear gradient of buffer A to buffer B (10 mM HEPES, 500 mM imidazole, 500 mM NaCl, pH 8.0).

### Protein identification of VHH OMV81-reactive bands: in-gel enzymatic digestion

Bands from *A. baumannii* 19606 and 17961 OMVs that were reactive with VHH OMV81 by western blot were excised at an equivalent molecular weight from a total protein silver-stained gel (see Western blotting below). The gel bands were first destained using a solution of 30 mM potassium ferricyanide and 100 mM sodium thiosulfate, followed by three washes with water and dehydration with 100% acetonitrile. Reduction with 10 mM dithiothreitol (DTT) in 50 mM ammonium bicarbonate (ABC) at 56°C and alkylation with 55 mM iodoacetamide in 50 mM ABC while protected from light were subsequently performed, followed by three additional washes with water and dehydration with 100% acetonitrile. Proteins were enzymatically digested with 30 μL of 10 μg/mL trypsin (Promega, Madison, WI) in 50 mM ABC overnight at 37°C. Peptides were eluted by sonicating tubes in a water bath for 5 min and stored at −20°C until mass spectrometry analysis.

### Protein identification of VHH OMV81-reactive bands: mass spectrometry

Peptides were diluted 1 in 10 with 0.1% (v/v) formic acid. For nano-liquid chromatography tandem mass spectrometry (nLC-MS/MS), 10 μL of acidified peptide solution was injected onto a PepMap100 (300 μm × 5 mm, 5 μm 100 Å) C18 Acclaim pre-column (Thermo Fisher Scientific) using an UltiMate 3000 high-performance liquid chromatography (HPLC) system (Dionex, Sunnyvale, CA) and subsequently separated using a BEH130C18 (100 μm × 10 cm, 1.7 Å) column (Waters, Milford, MA). Peptides were eluted with an increasing percentage of solvent B over 72 min: 1% for 1 min, 1–6% over 3 min, 6–25% over 48 min, 25–40% over 9 min, and 40–85% over 3 min. This was followed by a decrease from 85 to 1% solvent B over 1 min, and thereafter, the column was held at 1% solvent B for 7 min for re-equilibration. Solvent A was 0.1% formic acid in HPLC grade water, while solvent B was 0.1% formic acid in acetonitrile. Data-dependent acquisition was used with collision-induced dissociation. Resulting MS^2^ fragmentation spectra were searched against an in-house *A. baumannii* database using the Mascot search engine (Matrix Science, Boston, MA). A minimum of two identified peptides and an ion cut off score of 50 were applied.

### Expression and purification of recombinant CsuA/B

The full-length DNA sequence encoding CsuA/B from *A. baumannii* 19606 (Tomaras et al. 2008; NCBI Ref Seq. WP_000790104.1) was modified to include a C-terminal GAHHHHHH tag, synthesized and cloned into pET-24(+) using *Bam*HI and *Hind*III restriction sites (Twist Biosciences, South San Francisco, CA). *E. coli* BL21(DE3) cells were transformed with the resulting vector by electroporation and incubated in SOC medium at 37°C for 1 h. Cells were plated on Luria-Bertani (LB) agar plates containing 50 μg/mL kanamycin. A single colony was used to inoculate 3 mL of LB containing 50 μg/mL kanamycin, and the culture was grown overnight at 37°C with 250 rpm shaking. This culture was used to inoculate 1 L of LB containing 50 μg/mL kanamycin in a baffled flask, which was grown at 37°C with agitation at 250 rpm until an OD_600_ of 0.6 was reached. The culture was induced using 10 μM IPTG followed by incubation at 28°C overnight with 250 rpm shaking. The cells were then lysed by sonication in 160 mL of lysis buffer (10 mM HEPES, 500 mM NaCl, 20 mM imidazole, pH 8.0) containing 1 mM phenylmethylsulfonyl fluoride and centrifuged at 10,000 × *g* for 10 min. The supernatant was discarded, and the cell pellet was resuspended in denaturing urea buffer (50 mM sodium phosphate, 300 mM NaCl, 8 M urea, 5 mM imidazole, pH 8.0), sonicated and centrifuged at 12,000 × *g* for 20 min. The supernatant was collected and centrifuged again as above. Next, 500 μL of Ni Sepharose excel resin (Cytiva) was equilibrated in denaturing urea buffer and added to the supernatant, followed by mixing for 1 h at 4°C. The beads were drained, washed with 25 mL of denaturing urea buffer, and eluted with 10 mL of urea elution buffer (50 mM sodium phosphate, 300 mM NaCl, 8 M urea, 500 mM imidazole, pH 8.0). The eluate was collected and dialyzed for 24 h at 4°C in 1 L of PBS containing 0.1 mM DTT and then once more in PBS without DTT. The refolded protein was concentrated to 1 mL in a 10 kDa cutoff Amicon Ultra-15 centrifugal filter and purified by SEC using a Superdex 75 Increase 10/300 GL column (Cytiva) connected to an ÄKTA FPLC protein purification system with a PBS mobile phase.

### Intact LC-MS of recombinant CsuA/B

Purified CsuA/B was analyzed by intact LC-MS using an UltiMate 3000 HPLC system coupled *via* an Ion Max electrospray source to an LTQ-Orbitrap™ XL mass spectrometer (Thermo Fisher Scientific). Approximately 5 μg of protein was injected onto a 2.1 × 30 mm Poros R2 reverse phase column (Applied Biosystems, Waltham, MA) and desalted using a 3 min, 3 mL/min, 0.1% formic acid water/acetonitrile linear gradient (10–75% acetonitrile). The column and solvents were heated to 80°C to improve protein elution peak shape. The HPLC eluent was split to 100 μL/min before the electrospray source. The LTQ-Orbitrap™ XL instrument was tuned with intact myoglobin for optimal detection of small (<50 kDa) proteins, and scans were recorded at 400–2000 m/z and 15,000 FT resolution. For analysis, the mass spectra acquired across the protein elution peak were summed, and the ion envelope deconvoluted into a molecular weight profile using the MaxEnt 1 module of MassLynx (Waters).

### Surface plasmon resonance (SPR)

VHHs were purified by SEC using a Superdex 75 Increase 10/300 GL column connected to an ÄKTA FPLC protein purification system with HBS-EP buffer (10 mM HEPES, pH 7.4, 150 mM NaCl, 3 mM EDTA, 0.005% (v/v) surfactant P20) as the mobile phase. The binding kinetics of OMV81 interacting with recombinant CsuA/B were measured using a Biacore T200 instrument (Cytiva) at 25°C. A total of 205 response units (RU) of CsuA/B, 892 RU of recombinant mucin-1 (MUC1; produced in-house), and 275 RU of FC5 VHH (Haqqani et al. 2018) were immobilized on a Series S CM5 sensor chip through amine coupling in 10 mM acetate buffer, pH 4. OMV81, α-MUC1 (unpublished data), and α-SARS-CoV-2 spike (Rossotti et al. 2022) VHHs were flowed over all surfaces at a flow rate of 40 μL/min in HBS-EP buffer with 180 s of contact time and 180 s of dissociation time. The α-spike VHH was injected at concentrations ranging from 3.9 to 1000 nM, the α-MUC1 VHH was injected at concentrations ranging from 0.625 to 10 nM, and OMV81 VHH was injected at concentrations ranging from 2.5 to 40 nM over CsuA/B and from 3.9 to 1000 nM over the other surfaces. All surfaces were regenerated with 10 mM glycine, pH 1.5, for 60 s at 100 μL/min. Reference flow cell subtracted sensorgrams were fit to a 1:1 binding model and kinetics and affinities determined using BIAevaluation Software v3.2 (Cytiva).

### Indirect, whole-cell, and competitive ELISAs

For indirect ELISAs, wells of microtiter plates were coated with either 100 μL of 5 μg/mL OMV (for OMV SEC fractions, 100 μL of each fraction regardless of protein concentration) at 4°C for 72 h or 100 μL of 1 μg/mL recombinant CsuA/B overnight at 4°C. For whole-cell ELISAs, *A. baumannii* cells were streaked from frozen stocks on LB agar plates and incubated overnight at 37°C. A loop of the collected bacteria was vortexed in 0.9% PBS, then diluted to an OD_600_ of 0.1 into TSB medium, and grown overnight at 37°C. The cells were centrifuged at 10,000 × *g* for 10 min, supernatant decanted, and cells resuspended in PBS. The cells were then heated for 55 min at 56°C, centrifuged again and resuspended in fresh PBS to an OD_600_ of 1.0. *E. coli* BL21(DE3) cells were prepared identically using LB instead of TSB. Next, 100 μL of bacterial cells was plated in wells of a Nunc MaxiSorp flat-bottom microtiter plate (Invitrogen) and left to adhere at 4°C for 72 h. For competitive ELISAs, wells of microtiter plates were coated with 100 μL of 3.8 μg/mL 19606 OMV in PBS at 4°C for 72 h or 100 μL of 1 μg/mL recombinant CsuA/B overnight at 4°C.

For all ELISAs, wells were washed once with 200 μL of PBST, blocked for 1 h with 200 μL of 2.5% (w/v) bovine serum albumin in PBST (BSA-PBST), emptied, and incubated with various concentrations of VHH in BSA-PBST for 1 h at room temperature. For competitive ELISAs, 50 ng/mL or 25 ng/mL of VHH OMV81 was pre-incubated with recombinant CsuA/B at various concentrations for 30 min at room temperature prior to addition to the plate. The wells were then emptied, washed 4× with PBST, and incubated for 1 h with 100 μL of either HRP-conjugated rabbit anti-c-Myc IgG (Cat#A190-105P, Bethyl Laboratories) or rabbit anti-His_6_ IgG (Cat#A190-114P, Bethyl Laboratories) diluted 1:5000 in BSA-PBST. The wells were then washed 3× with PBST and 1× with PBS. Finally, 50 μL of High Sensitivity TMB ELISA Substrate was added to the wells and incubated at room temperature for 10 min. Development was stopped with 50 μL of 1 M phosphoric acid. Absorbance was measured at 450 nm.

### Western blotting

Samples of OMV, recombinant CsuA/B, and whole *A. baumannii* cells prepared as described above were diluted into Laemmli buffer containing 175 mM DTT. Samples were incubated at 95°C for 5 min, centrifuged, and electrophoresed in a 4–20% Mini-Protean TGX Stain-Free Protein Gel (Bio-Rad) using a Mini-Protean Tetra Vertical Electrophoresis Cell (Bio-Rad). Samples were then transferred onto Immobilon-P polyvinylidene fluoride (PVDF) membrane (MilliporeSigma). Membranes were washed once in PBST and blocked for 1 h at room temperature in BSA-PBST. Membranes were then incubated with VHH in BSA-PBST overnight at 4°C, washed 5× for 5 min in PBST, and then incubated with 1:5000 dilutions of HRP-conjugated rabbit anti-c-Myc IgG or rabbit anti-His_6_ IgG in BSA-PBST for 1 h at room temperature. Membranes were then washed as above, incubated for 1 min in ECL Pierce^TM^ Western Blotting Substrate (Thermo Fisher Scientific) and imaged on a ChemiDoc XRS+ Gel Imaging System (Bio-Rad). An equivalent gel was silver-stained for total protein and used for protein identification of VHH OMV81-reactive bands. Briefly, the proteins were fixed in the gel with 50% (v/v) ethanol and 5% (v/v) acetic acid, followed by two 10 min washes with water. The gel was sensitized using 0.02 N sodium thiosulfate, followed by two 5 min washes with water and subsequently incubated in 0.1 N silver nitrate for 30 min. Proteins were visualized with developing solution (0.04% (w/v) formaldehyde and 2% (w/v) sodium carbonate), and the reaction was quenched with 5% acetic acid. Western blot and silver-stained gel images were aligned to permit excision of VHH OMV81-reactive protein bands from the gel.

### Preparation of TAMRA-labeled VHH OMV81

OMV81 VHH bearing a free C-terminal Cys (39 nmol) was added to 390 nmol of Tris(2-carboxyethyl) phosphine (TCEP) in 1.5 mL of PBS (pH 7.4) and mixed for 1 h at room temperature. Next, 975 nmol of 5(6)TAMRA-C6-maleimide (Anaspec, Freemont, CA) in 10 μL of dimethyl sulfoxide was added to the solution. The sample was mixed, incubated in the dark at room temperature for 4 h, and then incubated for another 16 h at 4°C. Thereafter, 200 μL of Ni Sepharose excel resin equilibrated in PBS was added to the sample and mixed for 1 h. The resin was washed with 30 mL of PBS and eluted with 10 mL PBS containing 500 mM imidazole. The labeled VHH was then buffer exchanged back into PBS using a 3 kDa cutoff Amicon centrifugal filter.

### Fluorescence microscopy

For microscopy experiments, *A. baumannii* cells were grown as described above for whole-cell ELISA. The cell suspension (500 μL) was centrifuged, washed once in PBS, and then resuspended in 500 μL of PBS containing 10 μg/mL TAMRA-labeled VHH OMV81 and 20 μg/mL Hoechst 33342 (Cayman Chemical Company, Ann Arbor, MI). The sample was incubated for 30 min at room temperature protected from light, then centrifuged, and washed with PBS. The cells were centrifuged and resuspended in 4% formaldehyde solution for 1 h at room temperature, washed twice with PBS, and then suspended in 50 μL of Dako Fluorescence Mounting Medium (Agilent). Finally, 5 μL of the cell solution was placed on a glass coverslip, sealed, and imaged on an IX81 inverted microscope system (Olympus, Tokyo, Japan) at 600× magnification.

## Results

**Fig. 1:**
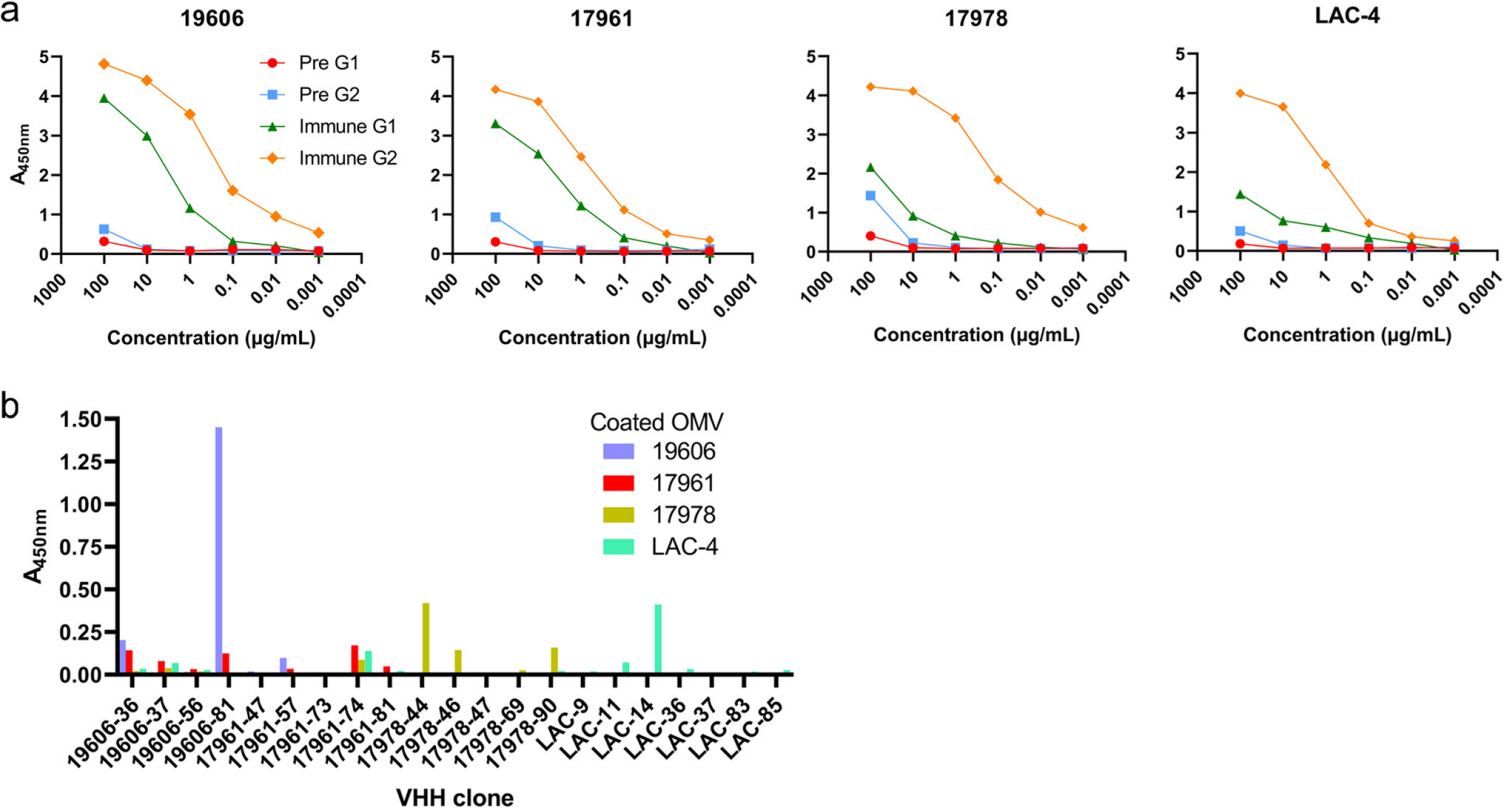
*A. baumannii* OMV llama immunization, serum response monitoring, and VHH isolation. **a** Pre-immune and immune (day 42) polyclonal serum IgGs from a llama immunized with OMVs from four *A. baumannii* strains were separated by protein G chromatography into heavy-chain-only IgG (G1; IgG2b) and conventional IgG (G2; IgG1) fractions. ELISA wells were coated with OMVs from each strain and binding of the G1 and G2 fraction polyclonal IgGs was detected with an HRP-conjugated anti-llama IgG. **b** Binding of VHHs isolated by panning a phage-displayed VHH library on OMVs. ELISA plates were coated with 3.8 μg/mL OMV from the indicated *A. baumannii* strain, probed with 10 μg/mL (~600–650 nM) of purified VHH monomer and detected with an HRP-conjugated anti-His_6_ IgG

OMVs were prepared from *A. baumannii* strains 19606, 17961, 17978, and LAC-4. Particle analysis revealed a monodispersed distribution with median particle size ranging from 199 to 249 nm, depending on the strain (Figure S1, Table S1). SEC revealed high-molecular weight species eluting in the Superose 6 column void volume and in the ~1000–500 kDa range, along with several species with molecular weight less than 60 kDa (Figure S2). The OMV preparations were not further purified (e.g., by density gradient centrifugation or gel filtration) and thus presumably contained extracellular materials such as flagella, fimbria, pili, and large protein complexes and/or aggregates. A llama was immunized using a mixture of all four OMV preparations. Pre-immune and immune sera were collected and fractionated on a protein G column to separate heavy-chain-only IgGs (G1 fraction; IgG2b; homodimer of single polypeptide chain consisting of VHH domain-hinge-constant heavy domains CH2-CH3) from conventional IgGs (G2 fraction; IgG1; dimer of polypeptide heterodimers each consisting of VH domain-CH1 domain-hinge-CH2-CH3 paired with a VL domain-constant light domain CL). Binding of polyclonal IgGs to OMVs was tested in ELISA. Binding of both conventional and heavy-chain polyclonal IgGs post immunization were consistently higher than the pre-immune controls, indicating the elicitation of antibodies against the OMV preparations (Fig. 1a). Heavy-chain G1 responses were stronger against OMVs from 19606 and 17961 strains compared to 17978 and LAC-4, while conventional G2 responses were similar for all OMVs. An immune phage-displayed VHH library was constructed and subjected to three rounds of panning against each of the four OMVs in parallel. Polyclonal phage ELISA from each panning round indicated enrichment for binding phages against the OMVs used for panning, as well as some level of cross reactivity of polyclonal phages panned against OMV 19606 and 17961 (data not shown). For each OMV panning campaign, 90 phagemid-containing *E. coli* TG1 colonies were selected at random, tested in monoclonal phage ELISA, and sequenced, yielding between 5 and 7 unique phage ELISA-positive VHH sequences per OMV strain. DNAs encoding these VHHs were synthesized and sub-cloned into an expression vector (pSJF2H), and the VHHs were expressed in *E. coli* TG1 cells and purified by (IMAC). The resulting VHHs were analyzed for binding against OMVs from all four *A. baumannii* strains by soluble ELISA. Of the VHHs tested, VHH clone 19606-81 (hereafter referred to as VHH OMV81; 10 ug/mL) gavethe highest signal against OMVs from strain 19606, which it was initially selected on, as well as slight cross reactivity towards OMVs from 17961 (Fig. 1b). Several other VHHs showed lower levels of binding to immobilized OMVs.

**Fig. 2:**
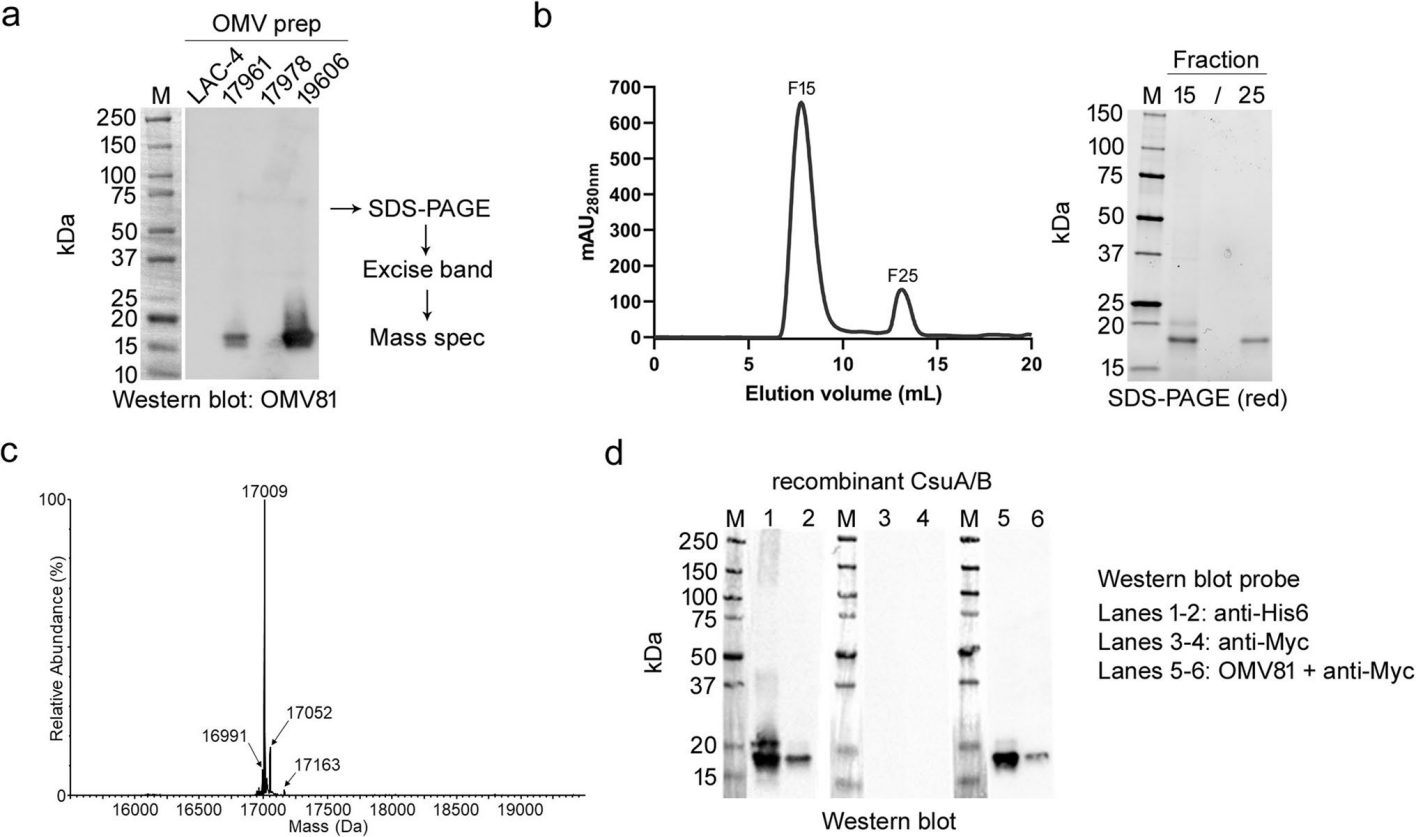
Identification of CsuA/B as the target of VHH OMV81 and recombinant CsuA/B production. **a** *A. baumannii* OMVs (5 μg) were separated by gel electrophoresis, transferred to a PVDF membrane and probed with 10 μg/mL (620 nM) VHH OMV81. VHH binding was detected with HRP-conjugated anti-c-Myc IgG. Bands on a corresponding SDS-PAGE gel were excised, digested, and subjected to bottom-up nLC-MS/MS for protein identification (*see* Table 2). **b**, c Production of recombinant His_6_-tagged CsuA/B in *E. coli*. b SEC chromatogram and reducing (red) SDS-PAGE analysis of IMAC-purified and refolded CsuA/B. **c** Confirmation by intact mass analysis. Molecular weight profile of the deconvoluted protein mass spectrum. The expected average mass for amino acids 24–186 of CsuA/B, including an internal disulfide bond, a GA linker, and His_6_ tag, is 17,009.71 Da. **d** Detection of recombinant CsuA/B with VHH OMV81. Cell extract (~1 μg) from *E. coli* BL21(DE3) cells expressing CsuA/B (lanes 1, 3, and 5) and 100 ng of purified recombinant CsuA/B (lanes 2, 4, and 6) were separated by SDS-PAGE and transferred to a PVDF membrane. Western blotting with an HRP-conjugated anti-His_6_ IgG bound directly to recombinant CsuA/B that contains a C-terminal GA-His_6_ tag (lanes 1–2). VHH OMV81 bound the identical band in a separate blot following the addition of an HRP-conjugated anti-c-Myc IgG (lanes 5–6). No binding was observed with the HRP-conjugated anti-c-Myc IgG alone (lanes 3–4)

To identify the antigen targeted by VHH OMV81, OMV preparations were separated by denaturing gel electrophoresis, and western blots were probed with the VHH (10 μg/mL). OMV81 bound predominantly to a single band present in both the 19606 and 17961 OMV samples, while binding to the other two OMVs (17978 and LAC-4) was not detectable (Fig. 2a), consistent with the binding patterns of the VHH ELISA. The western blot was used to guide the excision of the corresponding region of an SDS-PAGE gel containing the OMV sample. Peptides eluted from the gel following in-gel tryptic digestion were analyzed by bottom-up nLC-MS/MS for protein identification. A Mascot search of an in-house *A. baumannii* database identified several proteins, indicating co-migration of proteins with similar molecular weight on the SDS-PAGE gel. However, in both samples derived from 19606 and 17961 OMVs, the Csu pilus subunit protein CsuA/B was found to be the highest scoring protein (Table 2).

**Table 2:** Mass spectrometry-based protein identification of *A. baumannii* OMV bands detected by VHH OMV81 in western blot

Strain	Protein name	Accession number	Mascot score	Predicted mass (Da)	Peptide fragments identified
19606	CsuA/B	WP_000790104.1	34942	18563	5
	CsuB	WP_000876475.1	431	19440	2
	Hypothetical membrane protein	WP_032003135.1	360	38438	3
	Hemolysin	WP_032002807.1	258	15987	2
	Hypothetical membrane protein	WP_001037943.1	206	18699	2
	Hypothetical protein	WP_001211835.1	167	17321	2
17961	CsuA/B	WP_000790104.1	5811	18563	4
	Hemolysin	WP_032002807.1	889	15987	4
	Molecular chaperone GroEL	WP_001274623.1	800	57245	5
	Hypothetical protein	WP_000480885.1	667	15518	4
	Hypothetical membrane protein	WP_001097560.1	600	18095	4
	Hypothetical protein	WP_001211835.1	370	17321	3
	ATP synthase subunit B	WP_001024691.1	264	17037	2
	Hypothetical membrane protein	WP_032003135.1	228	38438	3
	Hypothetical protein	WP_001043188.1	184	15672	2
	Bacterioferritin	WP_001214863.1	183	18137	2
	Type II secretion system protein GspG	WP_000865304.1	180	20971	2
	Hypothetical membrane protein	WP_001037943.1	124	18699	2
	30S ribosomal protein S9	WP_000224786.1	80	14251	2

Given the strong evidence for the presence of CsuA/B in both the 19606 and 17978 OMV81-reactive bands, the full-length sequence of *A. baumannii* 19606 CsuA/B containing a C-terminal His_6_ tag was cloned into a pET-24(+) vector, and the resulting construct was used to transform *E. coli* BL21(DE3) cells for expression. The protein was found almost exclusively within inclusion bodies; however, recombinant CsuA/B was solubilized by sonication in denaturing urea buffer and purified by IMAC under denaturing conditions. The protein was refolded by dialysis over 24 h into PBS containing DTT and then further dialyzed into PBS to allow for the formation of intramolecular disulfide bridges. The protein was then passed over a SEC column as a final purification step and analyzed by SDS-PAGE (Fig. 2b). Proteins with the expected mass of CsuA/B by SDS-PAGE were observed in an earlier fraction (F15) and a later fraction (F25). Due to its higher purity and the likelihood of the earlier (F15) fraction corresponding to aggregated protein, the later fraction (F25) was used in all subsequent experiments. The mass of the SEC-purified recombinant CsuA/B was confirmed *via* intact mass spectrometry (Fig. 2c). The first 23 amino acids of the CsuA/B sequence were found to be missing, consistent with computational predictions of cleavage of the signal peptide sequence of this protein (Pakharukova et al. 2015). By western blotting, purified recombinant CsuA/B was recognized by both an anti-His_6_-antibody and by VHH OMV81, confirming the interaction between the VHH and CsuA/B (Fig. 2d). Furthermore, western blotting of *E. coli* BL21(DE3) cell lysates expressing recombinant CsuA/B showed almost no observable binding of VHH OMV81 to any additional protein bands other than CsuA/B.

**Fig. 3:**
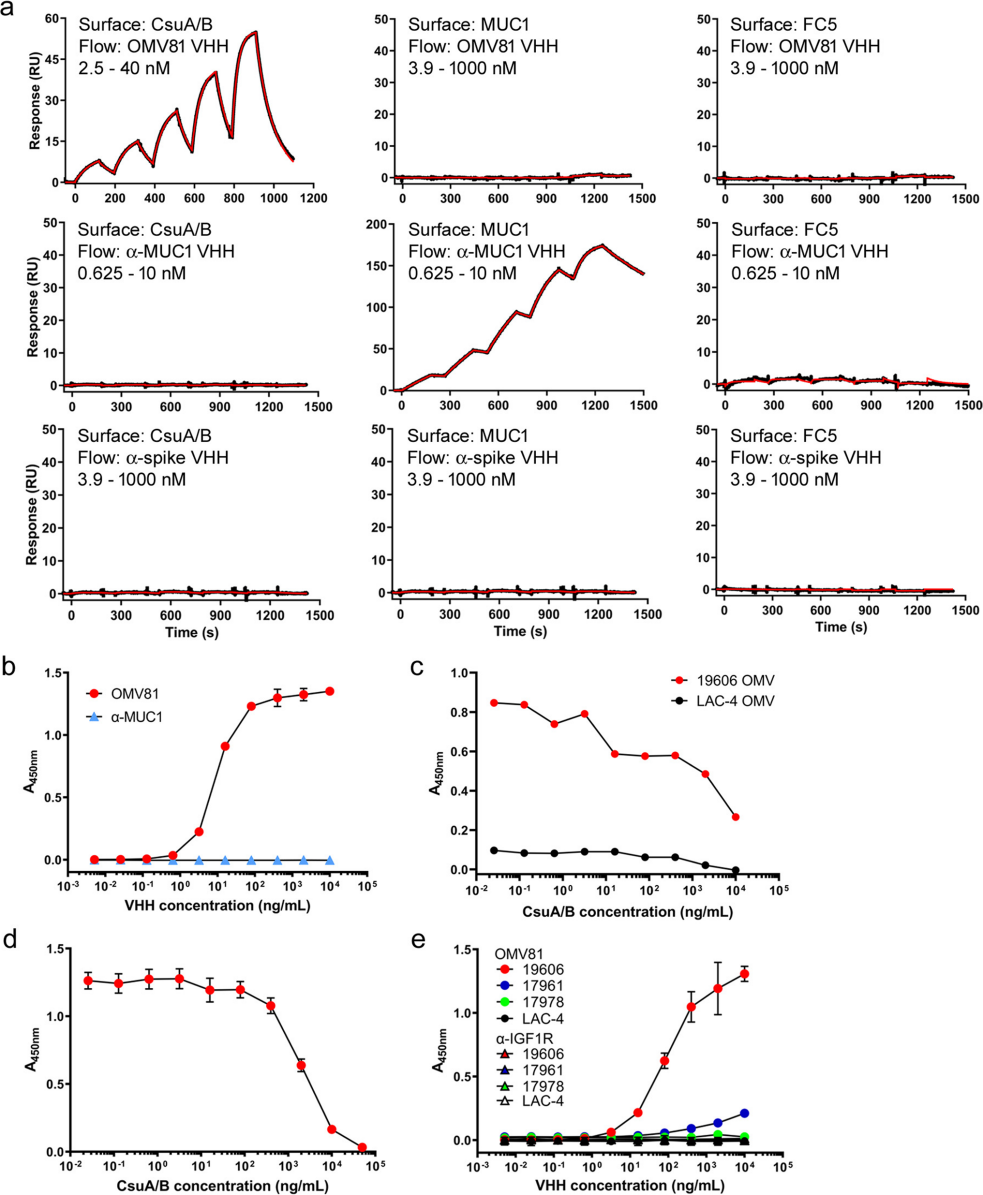
Validation of VHH OMV81 binding to recombinant CsuA/B. **a** SPR sensorgrams from OMV81 and control antibodies flowed over immobilized CsuA/B, MUC1, and FC5 surfaces. Black lines show raw data, and red lines show 1:1 binding model fits. Reported affinities and kinetics are from 3 independent experiments. **b** ELISA responses from dilutions of VHH OMV81 and an anti-MUC1 VHH binding to immobilized recombinant CsuA/B. **c**, **d** Competitive ELISAs of recombinant CsuA/B and VHH OMV81. Microtiter wells were coated with **c** OMVs from 19606 and LAC-4 (3.8 μg/mL) or **d** recombinant CsuA/B (1 μg/mL) and treated with **c** 50 ng/mL (3.2 nM) or **d** 25 ng/mL (1.6 nM) VHH OMV81 pre-incubated for 30 min with increasing concentrations of recombinant CsuA/B. **e** ELISA responses from dilutions of VHH OMV81 and an anti-IGF1R VHH control (Alata et al. 2022) binding to coated *A. baumannii* OMVs. Error bars represent the mean ± SEM from *n* = 3 experimental replicates

Once the binding partner of VHH OMV81 was confirmed to be CsuA/B, the kinetics and affinity of the interaction were determined by SPR. Recombinant CsuA/B was immobilized on a sensor chip alongside two irrelevant control proteins, human MUC1 and FC5, a blood–brain barrier-transmigrating VHH (Haqqani et al. 2018). OMV81, an anti-MUC1 VHH (unpublished data), and an irrelevant VHH specific to the SARS-CoV-2 spike protein (Rossotti et al. 2022) were flowed over all immobilized proteins. VHH OMV81 bound specifically to CsuA/B, the MUC1 VHH bound specifically to MUC1, and the control anti-spike VHH did not bind any surface as expected (Fig. 3a). The equilibrium dissociation constant (*K*_D_) of the interaction between OMV81 and CsuA/B was 16.7±0.1 nM (*k*_a_ = 7.32×10^5^ M^−1^ s^−1^, *k*_d_ = 1.22×10^−2^ s^−1^). Binding was further confirmed by ELISA against immobilized recombinant CsuA/B (Fig. 3b), where OMV81 bound CsuA/B with an *EC*_50_ of 9.7 nM, correlating well with the SPR data. ELISAs using 19606 OMV SEC fractions revealed the OMV81 VHH only recognized the high-molecular weight species eluting in the column void volume and in the ~1000–500 kDa range but not the low molecular weight species (Figure S2). In addition, pre-incubation of VHH OMV81 with recombinant CsuA/B inhibited binding to immobilized OMVs derived from 19606 (Fig. 3c) and to immobilized recombinant CsuA/B (Fig. 3d) in competitive ELISAs. VHH OMV81 binding to immobilized OMVs by ELISA mirrored the results of western blotting, with high binding of the VHH to 19606, weak binding to 17961 and no binding observed to 17978 or LAC-4 OMVs (Fig. 3e).

**Fig. 4:**
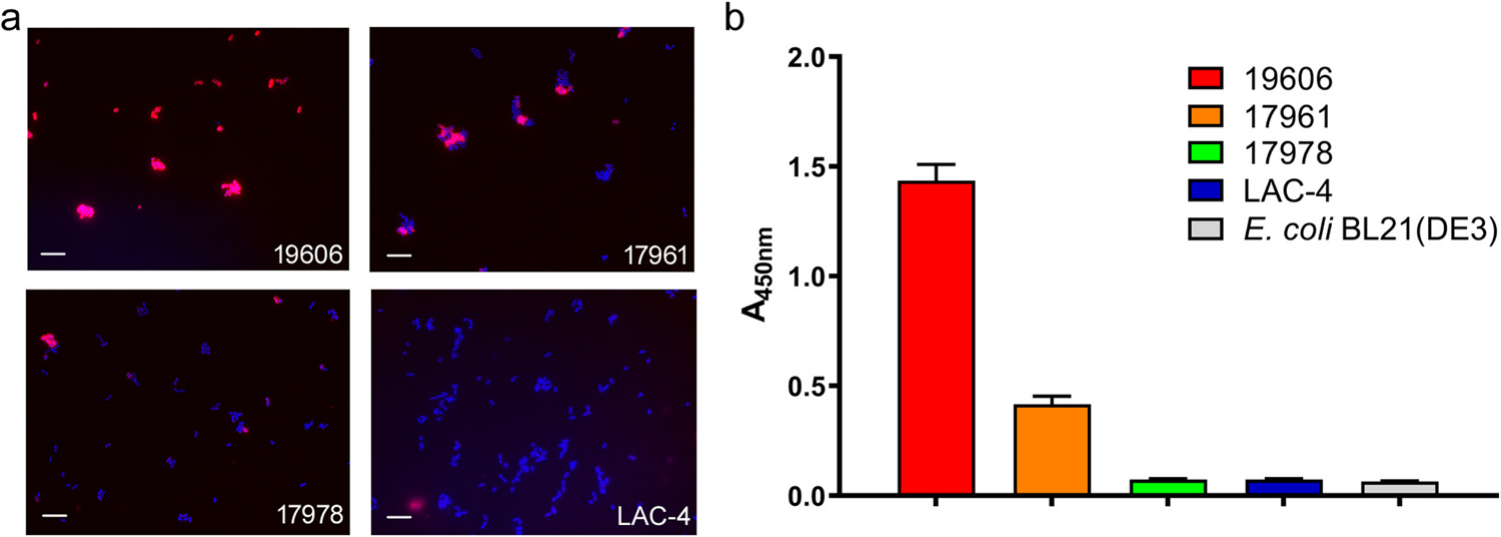
Binding of VHH OMV81 to the surface of *A. baumannii* cells. **a** *A. baumannii* cells were stained with 10 μg/mL (620 nM) of fluorescently labeled VHH OMV81 co-stained with Hoechst 33342. Bacteria were then fixed with formaldehyde and imaged on a glass coverslip. Hoechst fluorescence is shown in blue, and OMV81 fluorescence is shown in red/magenta. Scale bars (10 μm) are shown in white. **b** Whole cell ELISA of *A. baumannii* strains and VHH OMV81. *A. baumannii* and *E. coli* cells were grown overnight and heat killed. Bacterial samples with an OD_600_ of 1.0 were adhered to microtiter wells and probed with 10 μg/mL (620 nM) OMV81. Error bars represent the mean ± SEM from *n* = 3 experimental replicates

Next, we examined if VHH OMV81 could bind *A. baumannii* cells. A modified VHH was generated containing a free C-terminal cysteine and then fluorescently labeled with 5(6)-TAMRA *via* maleimide chemistry. *A. baumannii* cells representing the four strains from which OMVs were prepared and used in the initial immunization were grown, stained with TAMRA-OMV81 (10 μg/mL), and then fixed with formaldehyde. Visible cell staining was observed for *A. baumannii* 19606 and 17961, while weak or undetectable staining was observed for 17978 and LAC-4 (Fig. 4a). The 19606 strain was the only one that showed consistent cell staining for all bacteria in the field of view, while the other two strains (17961 and 17978) appeared to have more heterogeneous staining between individual cells. Staining of LAC-4 was not observed at a level discernable from an untreated control (Figures S3-S6).

Microscopy results were further confirmed by whole-cell ELISA using the *A. baumannii* strains. In this case, both 19606 and 17961 were bound by VHH OMV81 (10 μg/mL), while 17978 and LAC-4 showed similar binding as control *E. coli* BL21(DE3) cells (Fig. 4b). Taken together, these results indicated VHH OMV81 bound CsuA/B expressed on the surface of *A. baumannii* 19606 cells, potentially recognized CsuA/B on the surface of 17961 cells more weakly, and showed marginal staining of 17978 by microscopy despite no detectable binding in ELISA or western blot to OMVs or whole cells.

## Discussion

In this work, we immunized a llama with OMV preparations from several *A. baumannii* strains with a goal to isolate novel VHHs. VHHs were generated by panning the phage-display library on immobilized OMV preparations, which likely included extracellular material, with the antigen binding partner of one VHH, OMV81, being identified and characterized. The antigen targeted by OMV81 was CsuA/B, the major structural pilin protein of Csu pili in *A. baumannii* (Pakharukova et al. 2018). To our knowledge, this is the first defined antibody reagent identified against this protein. The OMV preparations used in this study may have contained large extracellular components (e.g., flagella, fimbria, pili, and other membrane-associated proteins) in addition to the OMVs; therefore, OMV81 may have been elicited by free Csu pili rather than OMV-embedded pili. Separation of 19606 OMV preparations by SEC followed by ELISA showed binding of the OMV81 VHH to high-molecular weight species which may represent either or both of OMVs and intact pili, including Csu pili.

As the major repeating structural subunit of the Csu pilus, CsuA/B is present in high copy number on the surface of bacteria bearing Csu pili, explaining the strong response when tested in ELISA against OMV preparations or whole cells. Comparative sequence analysis of *A. baumannii* strains 19606, 17961, 17978, and LAC-4 using BlastP indicated the presence of gene products corresponding to the sequence of CsuA/B in all strains except LAC-4; the CsuA/B amino acid sequences in these three strains had >99% sequence identity (Figure S7), while no CsuA/B homologs with >50% sequence identity are present in LAC-4. Interestingly, the 17978 strain was not found to produce detectable levels of CsuA/B as measured by OMV ELISA, whole-cell ELISA, or by western blotting of OMVs when performed alongside 19606 and 17961 strains; however, some weak binding was observed by fluorescence microscopy. Previous work has indicated that Csu pili are produced by 17978, although their expression may be affected by plasmids which are not stable under laboratory culture conditions and are affected by nutrient availability (Chen et al. 2022; Moon et al. 2017). Our experiments used *A. baumannii* grown to stationary phase for cell binding analysis and OMV production, which could have affected Csu pilus expression in certain strains. As such, it is possible that the culture conditions used in our experiments did not induce production of Csu pili in 17978 and 17961 at levels comparable to 19606. There may be other explanations of the differential strain reactivity of OMV81 VHH as well, but the presence or absence of a CsuA/B gene is clearly not the sole predictor of OMV81 VHH binding.

Csu pili are a primary contributor to the ability of *A. baumannii* to adhere to abiotic surfaces and are also associated with the production of biofilms and antibiotic resistance (Moon et al. 2017; Pakharukova et al. 2018; Romero et al. 2022). Association between biofilm production and CsuA/B expression may suggest that Csu pili would be expressed in biofilm-producing clinical isolates. Direct associations between Csu pilus expression and biofilm production have been observed in vitro (Pakharukova et al. 2018; Tomaras et al. 2003); however, expression of Csu pili is not required for the formation of biofilms in *A. baumannii* (Tomaras et al. 2008), suggesting that alternative and/or redundant pathways exist for biofilm production. In clinical infections, the Csu operon is present in nearly all biofilm-producing strains (Azizi et al. 2016; Zeighami et al. 2019); however, whether the protein is expressed in vivo and under what conditions remains to be elucidated.

Immunization of llamas with bacterial OMV preparations was successful in inducing an immune response against proteins associated with the outer membrane of *A. baumannii* cells. A strong polyclonal heavy-chain IgG response was observed against 19606 and 17961 OMV preparations. The weaker but measurable response generated against 17978 and LAC-4 OMV preparations suggests there could be other non-CsuA/B targets recognized by the heavy-chain IgGs. To the best of our knowledge, this is the first example of an OMV immunization in large mammals for antibody isolation. However, only one llama was used, and the VHH we isolated and characterized was found to recognize a pilus which may have been shed from the surfaces of cells and/or OMVs. Therefore, the general applicability of the approach is unclear at present and this is a limitation of our study.

OMV-based immunizations have been previously used in mice to produce polyclonal antibodies against *A. baumannii* (Huang et al. 2019) and in rabbits to produce monoclonal antibodies targeting phosphorylcholine on the surface of *Treponema pallidum* (Blanco et al. 2005). OMV-based immunization may in theory allow for the rapid development of VHHs that bind to outer membrane proteins in a natural context, which is important because many membrane-associated proteins, and especially those with multipass transmembrane domains, are difficult to express or isolate in their native form for immunization. Additionally, OMV immunization and panning may allow for the preservation of binding interactions between proteins and other membrane architecture which would prevent the isolation of antibodies against epitopes not normally exposed in vivo. The tradeoff of this strategy is the need for antigen identification after a VHH has been identified. In this study, we used western blotting, SDS-PAGE, in-gel digestion, and mass spectrometry to identify the target protein bound by a VHH elicited by immunization with OMV preparations. The antigen targeted by VHH OMV81 (CsuA/B) was subsequently confirmed through binding to a recombinant version of the protein, whole-cell ELISA, and microscopy. VHH OMV81 showed strong reactivity in denaturing western blots suggesting the VHH recognizes a linear or unstructured epitope. It is possible that other VHHs isolated using the OMV-based immunization strategy may bind conformational epitopes, limiting their ability to recognize linearized epitopes in western blot. For these types of VHHs, the target proteins may still be identified by native western blotting, or by immunoprecipitation with OMVs or full bacterial cells.

In conclusion, we have shown that a llama immunized with *A. baumannii* OMV preparations produced an anti-OMV preparation heavy-chain IgG response, allowing for the isolation of VHHs targeting bacterial surface proteins. For one VHH, the target was subsequently identified by mass spectrometry as CsuA/B. This approach may allow for the rapid identification of immunogenic membrane antigens from bacterial species for which proteomic studies of membrane composition may not be available. CsuA/B-targeting VHHs may also be useful as immunotherapeutics for treating *A. baumannii* infections or suitable as diagnostic agents; however, the breadth of strain reactivity needs to be considered.

## Supplementary information

ESM 1 (PDF 896 kb)

## Data Availability

All data generated or analyzed during this study are included in the article or available upon request.
