# Supplementary material for: Isolation and characterization of a VHH targeting the Acinetobacter baumannii cell surface protein CsuA/B
Source: Appl Microbiol Biotechnol. 2023 Jun 7;107(14):4567–80. doi: 10.1007/s00253-023-12594-1 (PMC10246537; doi:10.1007/s00253-023-12594-1)
Supplement: Supplementary file 1 — (PDF 896 kb) [file 253_2023_12594_MOESM1_ESM.pdf]

Supplemental Information

Isolation and Characterization of a VHH Targeting the *Acinetobacter baumannii* Cell Surface Protein CsuA/B

Eric K. Lei[1], Shannon Ryan[1], Henk van Faassen[1], Mary Foss[1], Anna Robotham[1], Isabel Baltat[1], Kelly Fulton[1], Kevin A. Henry[1,2], Wangxue Chen[1,3], Greg Hussack[1]

[1]Human Health Therapeutics Research Centre, National Research Council Canada, Ottawa, Ontario, Canada

[2]Department of Biochemistry, Microbiology and Immunology, University of Ottawa, Ottawa, Ontario, Canada

[3]Department of Biology, Brock University, St. Catharines, Ontario, Canada

Correspondence: Greg.Hussack@nrc-cnrc.gc.ca

Supplementary Table 1. Median OMV diameter and concentration as measured by NTA analysis. Concentration corresponds to the number of nanoparticles per mL of a 0.1 mg/mL sample of OMV, as determined by BCA analysis of protein concentration.

**Strain**	**Median diameter (nm)**	**Concentration (particles/mL)**
ATCC 19606	198.7	2.07E+9
ATCC 17978	215.7	7.50E+8
ATCC 17961	249.3	1.59E+10
LAC-4	217.0	2.85E+9

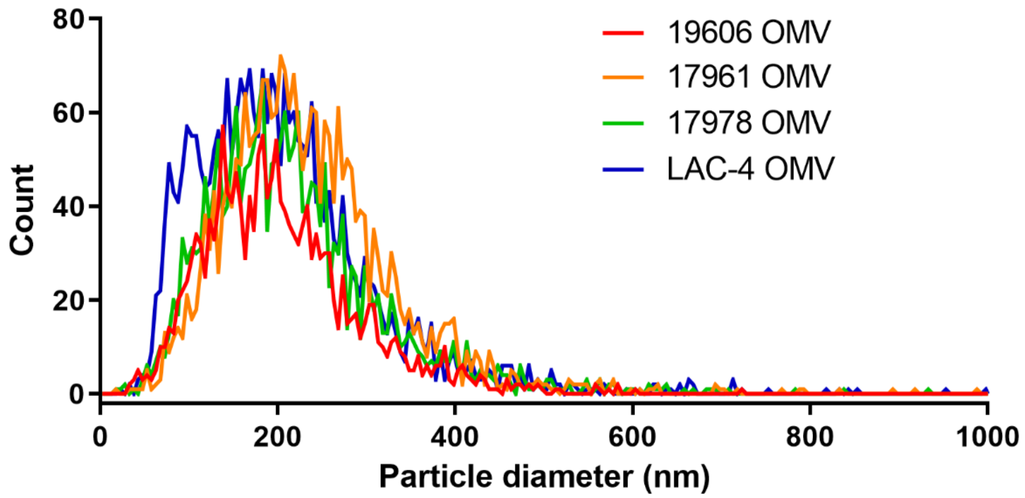

Supplementary Fig. 1 Analysis of purified OMVs using a Particle Metrix ZetaView Quatt PMX-420 instrument. Median particle sizes are shown in Supplementary Table 1.

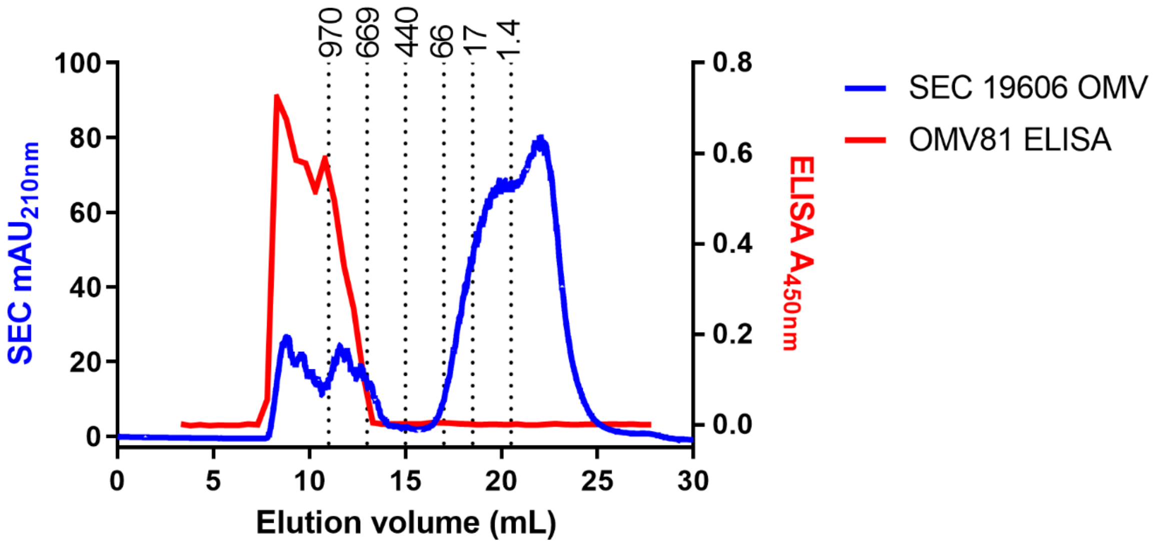

Supplementary Fig. 2 Size exclusion chromatogram of purified 19606 OMVs and ELISA performed on eluted fractions. Gel filtration was performed on a Superose 6 Increase 10/300 GL column. Protein standards (in kDa) are shown with dashed vertical lines. Eluted fractions were coated on ELISA wells and probed with 10 µg/mL OMV81 VHH (protocol described in Methods).

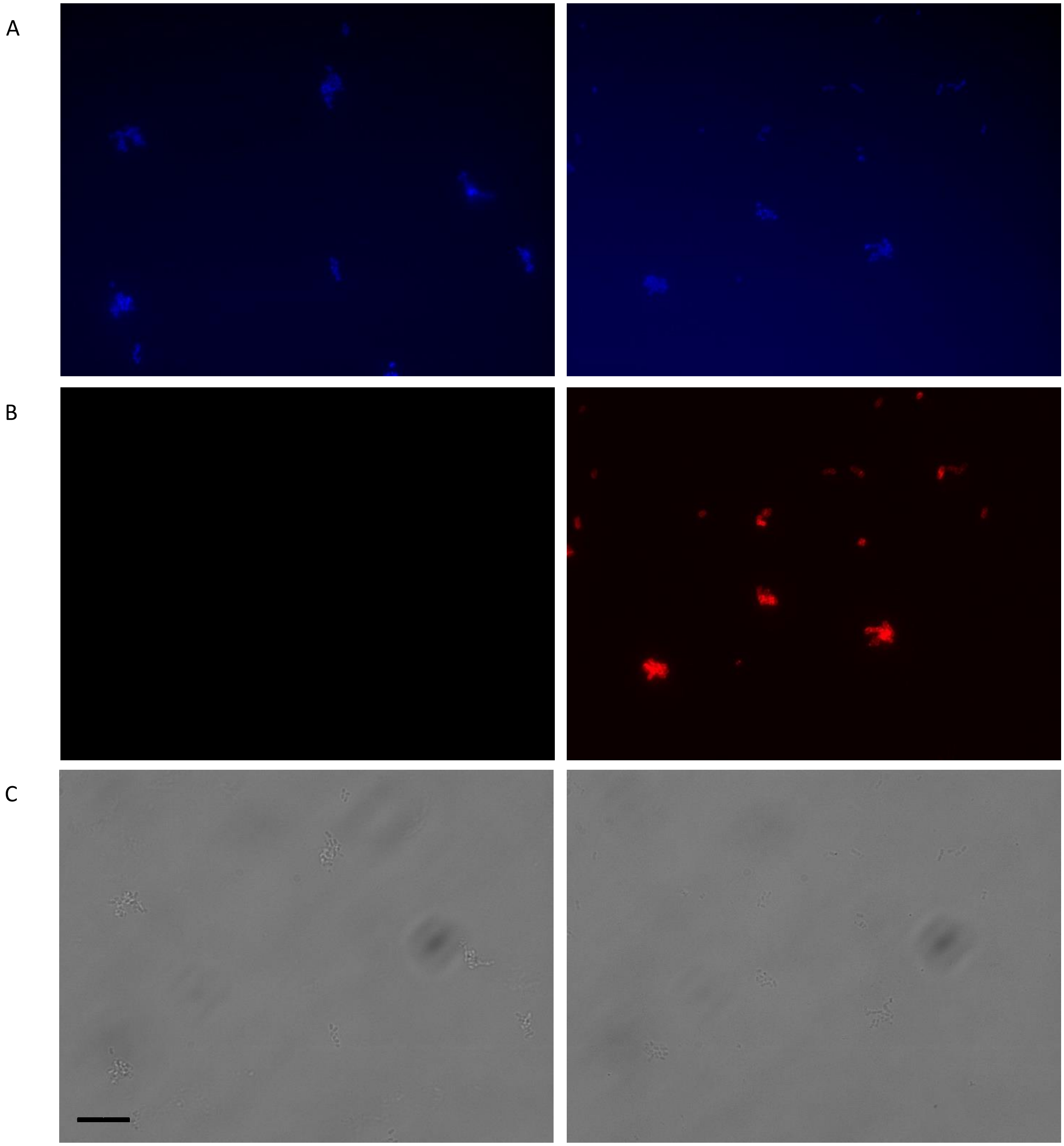

Supplementary Fig. 3 Fluorescence microscopy of *A. baumannii* ATCC 19606. Cells were stained with 20 µg/mL Hoechst 33342 and either left unstained by antibody (left column) or co-stained with 10 µg/mL of fluorescently labelled VHH OMV81 (right column). Hoechst 33342 fluorescence is shown in blue (A), OMV81 fluorescence is shown in red (B), and bright field is shown in grey (C). Black scale bar = 10 µm.

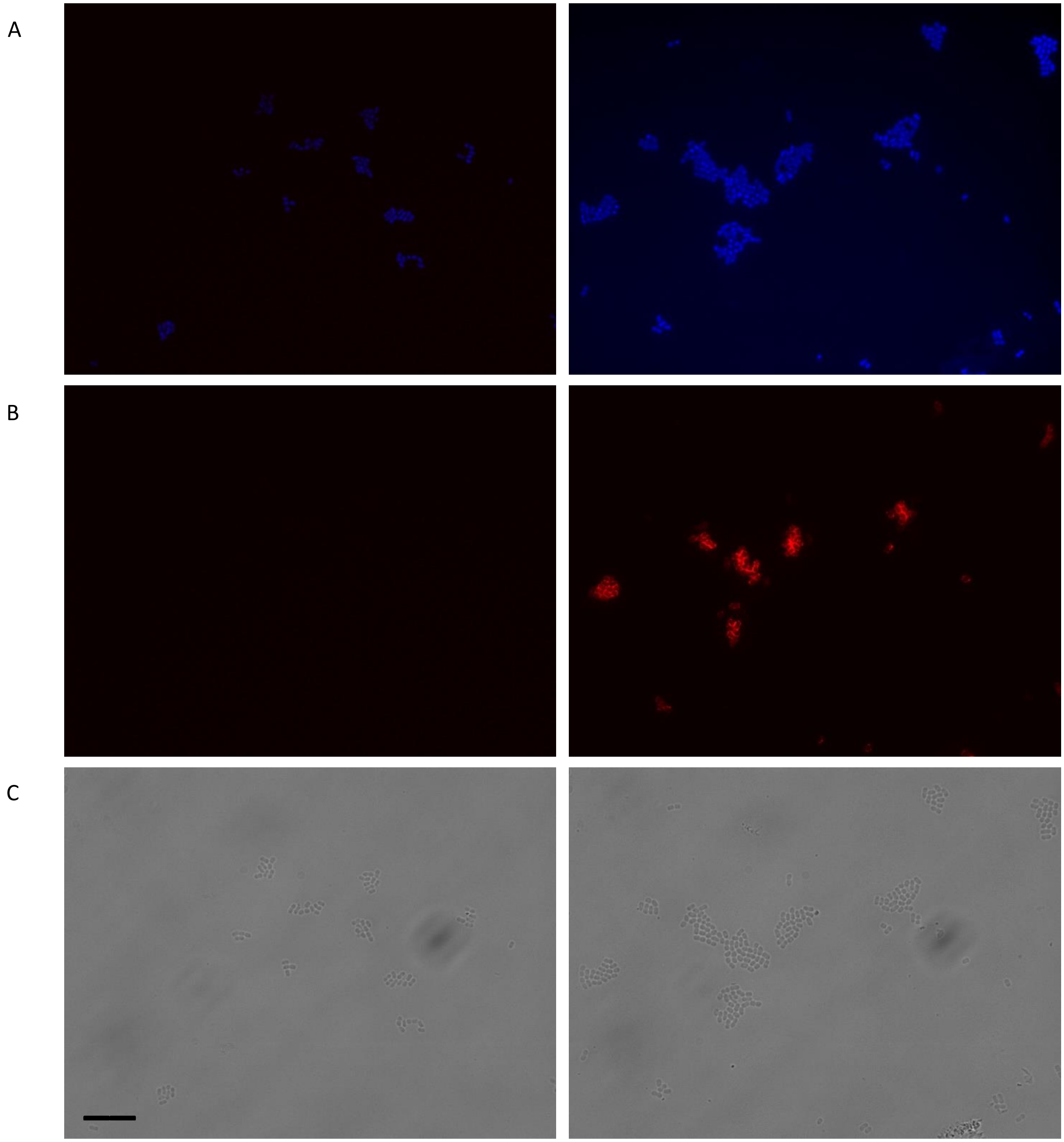

Supplementary Fig. 4 Fluorescence microscopy of *A. baumannii* ATCC 17961. Cells were stained with 20 µg/mL Hoechst 33342 and either left unstained by antibody (left column) or co-stained with 10 µg/mL of fluorescently labelled VHH OMV81 (right column). Hoechst 33342 fluorescence is shown in blue (A), OMV81 fluorescence is shown in red (B), and bright field is shown in grey (C). Black scale bar = 10 µm.

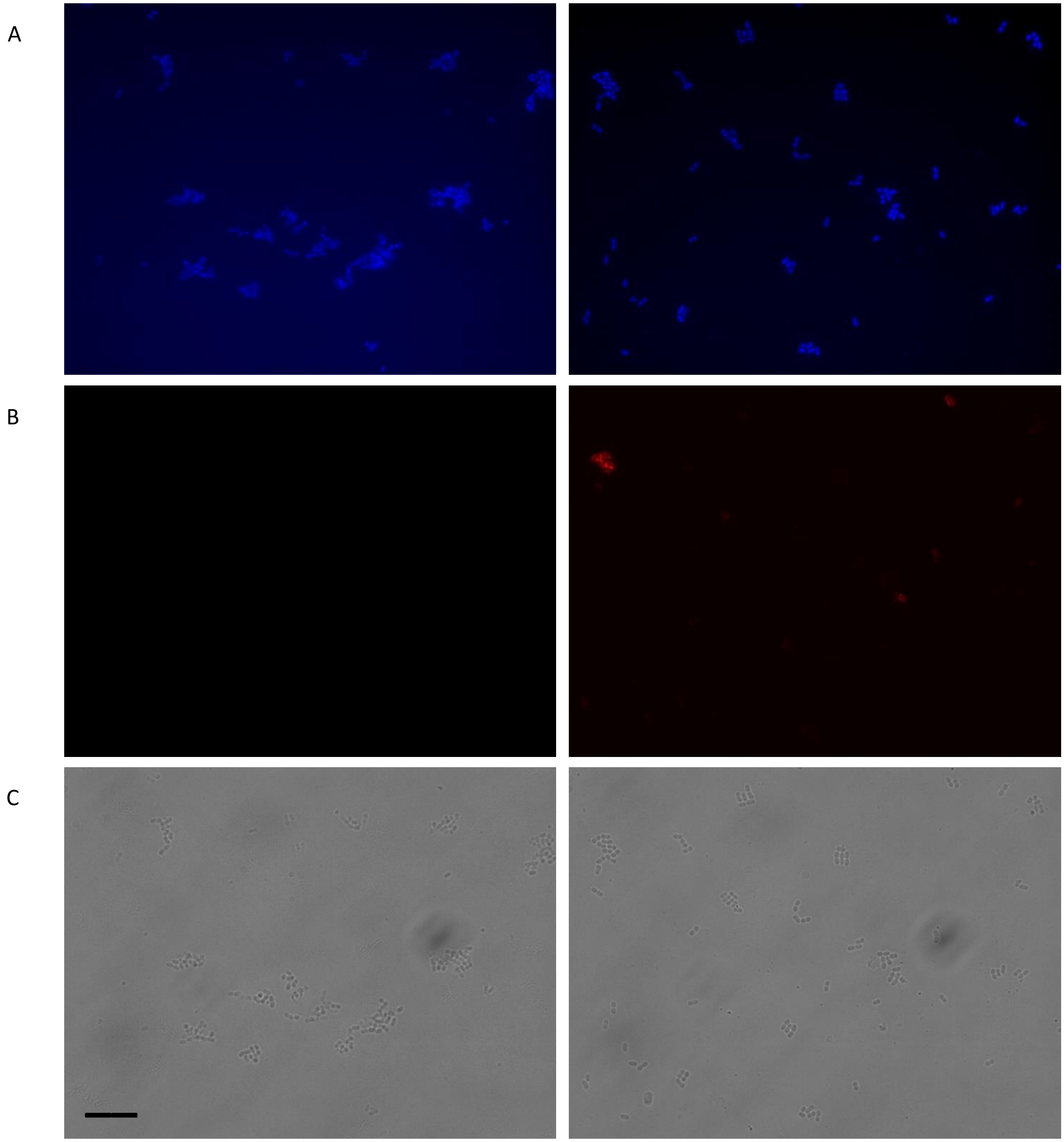

Supplementary Fig. 5 Fluorescence microscopy of *A. baumannii* ATCC 17978. Cells were stained with 20 µg/mL Hoechst 33342 and either left unstained by antibody (left column) or co-stained with 10 µg/mL of fluorescently labelled VHH OMV81 (right column). Hoechst 33342 fluorescence is shown in blue (A), OMV81 fluorescence is shown in red (B), and bright field is shown in grey (C). Black scale bar = 10 µm.

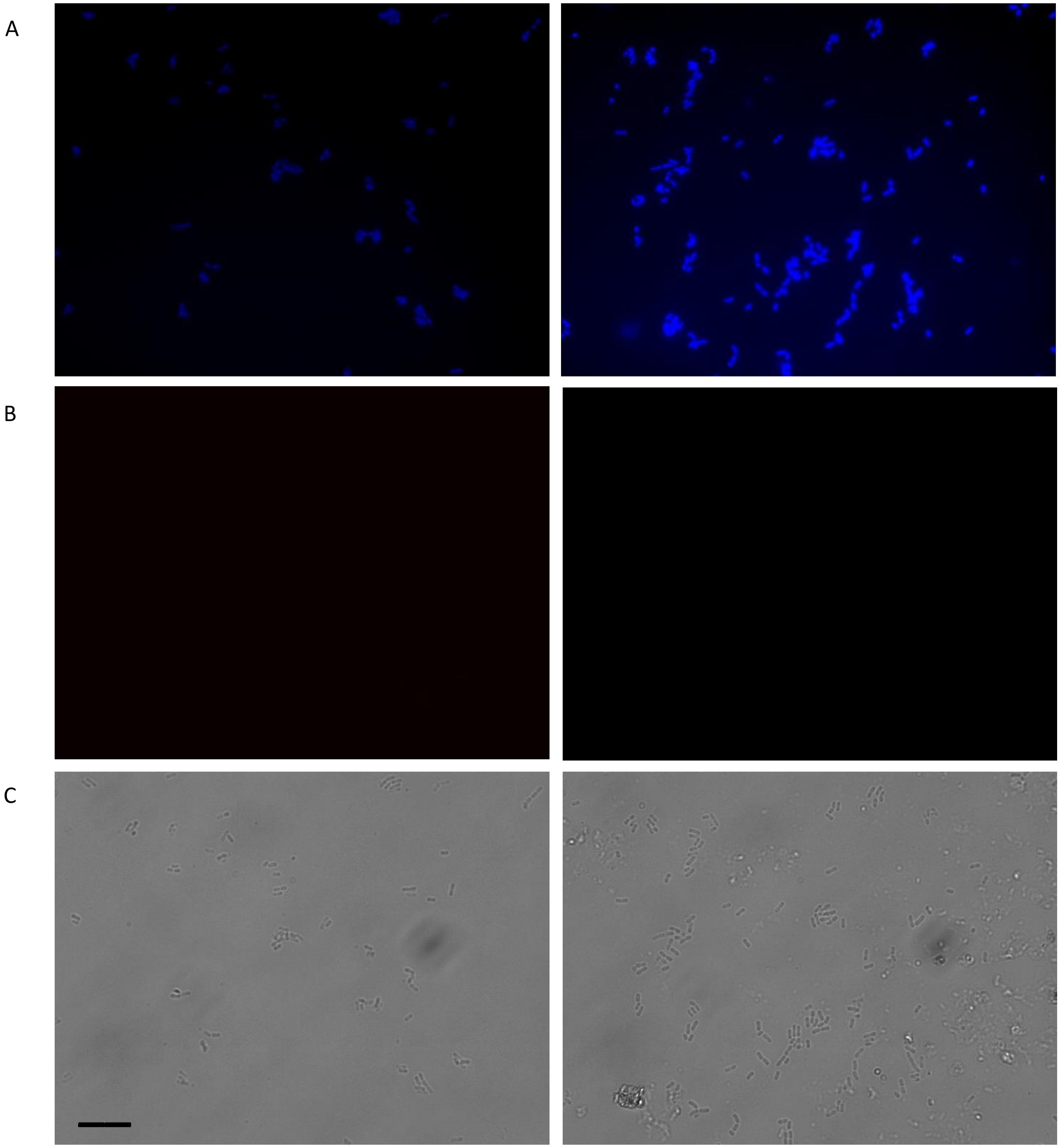

Supplementary Fig. 6 Fluorescence microscopy of *A. baumannii* LAC-4. Cells were stained with 20 µg/mL Hoechst 33342 and either left unstained by antibody (left column) or co-stained with 10 µg/mL of fluorescently labelled VHH OMV81 (right column). Hoechst 33342 fluorescence is shown in blue (A), OMV81 fluorescence is shown in red (B), and bright field is shown in grey (C). Black scale bar = 10 µm.

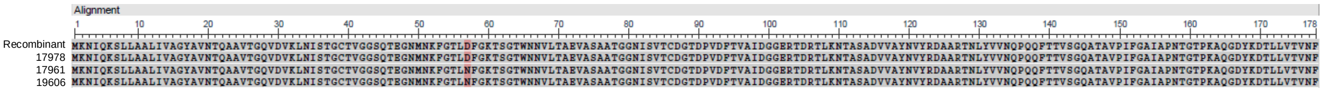

Supplementary Fig. 7 Protein sequence alignment for the recombinant CsuA/B sequence (*top row*) against CsuA/B from *A. baumannii* strains ATCC 17978 (CP049363.1), ATCC 17961 (CP046654.1), and ATCC 19606 (CP065432.1), respectively. Residues not identical between the four sequences are highlighted in red. No CsuA/B sequence with >50% identity was found for *A. baumannii* strain LAC-4.
